# Supplementary material for: Urban walkability through different lenses: A comparative study of GPT-4o and human perceptions
Source: PLoS One. 2025 Apr 29;20(4):e0322078. doi: 10.1371/journal.pone.0322078 (PMC12040139; doi:10.1371/journal.pone.0322078)
Supplement: S2 Table — (DOCX) [file pone.0322078.s002.docx]

**S2 Table.** *GPT-4V responses to paired images.*

| Perception | Images number | Number of responses out of 15 | |
| --- | --- | --- | --- |
|  |  | Image 1 | Image 2 |
| Overall walkability | 44,1 | 5 | 10 |
|  | 77,19 | 15 | 0 |
|  | 28,94 | 0 | 15 |
|  | 48,12 | 0 | 15 |
|  | 27,45 | 0 | 15 |
|  | 69,54 | 0 | 15 |
|  | 24,97 | 10 | 5 |
|  | 6,39 | 15 | 0 |
| Feasibility | 60,56 | 0 | 15 |
|  | 105,26 | 15 | 0 |
|  | 98,14 | 15 | 0 |
|  | 65,68 | 0 | 15 |
|  | 23,16 | 0 | 15 |
|  | 37,21 | 15 | 0 |
|  | 40,82 | 0 | 15 |
|  | 74,80 | 4 | 11 |
| Accessibility | 53,100 | 6 | 9 |
|  | 75,83 | 0 | 15 |
|  | 30,2 | 0 | 15 |
|  | 66,50 | 11 | 4 |
|  | 46,51 | 15 | 0 |
|  | 22,57 | 0 | 15 |
|  | 10,89 | 15 | 0 |
|  | 38,55 | 6 | 9 |
| Safety | 64,3 | 0 | 15 |
|  | 35,17 | 15 | 0 |
|  | 42,91 | 0 | 15 |
|  | 86,104 | 15 | 0 |
|  | 34,52 | 0 | 15 |
|  | 85,63 | 15 | 0 |
|  | 31,79 | 0 | 15 |
|  | 29,92 | 0 | 15 |
| Comfort | 103,87 | 15 | 0 |
|  | 95,101 | 0 | 15 |
|  | 11,43 | 6 | 9 |
|  | 73,9 | 15 | 0 |
|  | 49,62 | 5 | 10 |
|  | 4,71 | 0 | 15 |
|  | 13,99 | 0 | 15 |
|  | 76,93 | 15 | 0 |
| Liveliness | 8,67 | 15 | 0 |
|  | 41,81 | 0 | 15 |
|  | 15,90 | 0 | 15 |
|  | 18,20 | 0 | 15 |
|  | 61,70 | 0 | 15 |
|  | 36,33 | 15 | 0 |
|  | 106,47 | 0 | 15 |
|  | 78,5 | 15 | 0 |
