# Supplementary figures and images for: Urban walkability through different lenses: A comparative study of GPT-4o and human perceptions
Source: PLoS One. 2025 Apr 29;20(4):e0322078. doi: 10.1371/journal.pone.0322078 (PMC12040139; doi:10.1371/journal.pone.0322078)

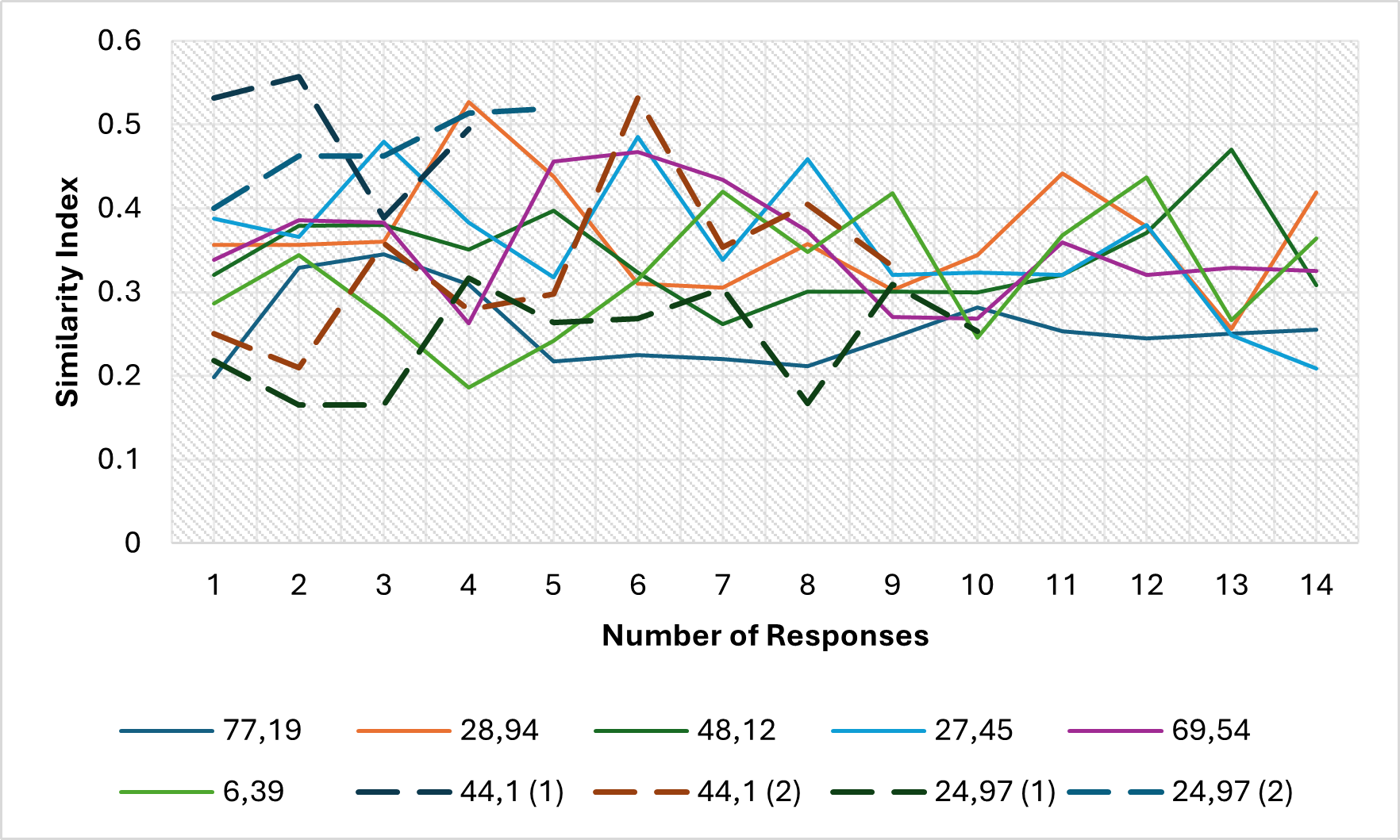

Supplement: S1 Fig — (TIF) [file pone.0322078.s003.tif]

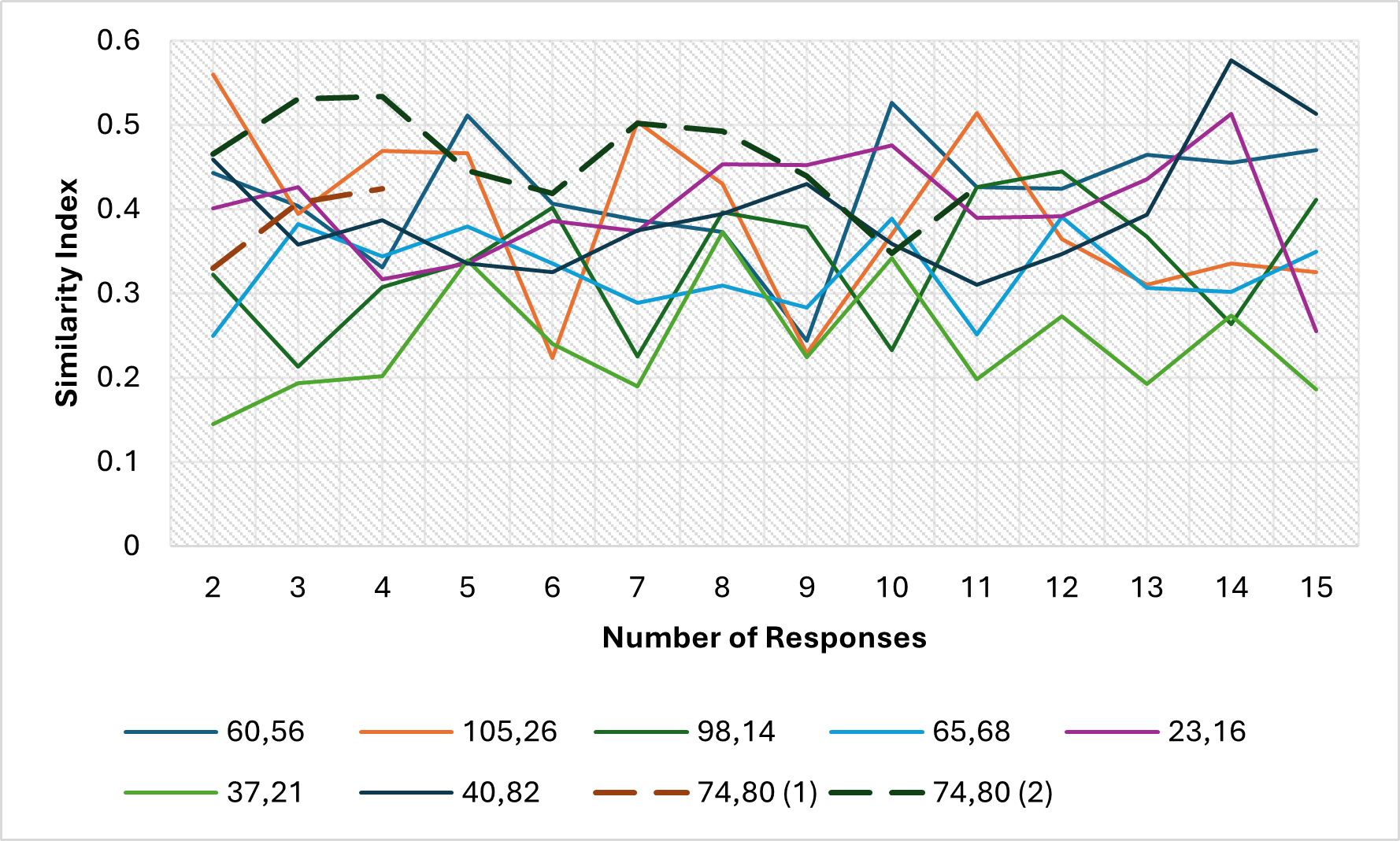

Supplement: S2 Fig — (TIF) [file pone.0322078.s004.tif]

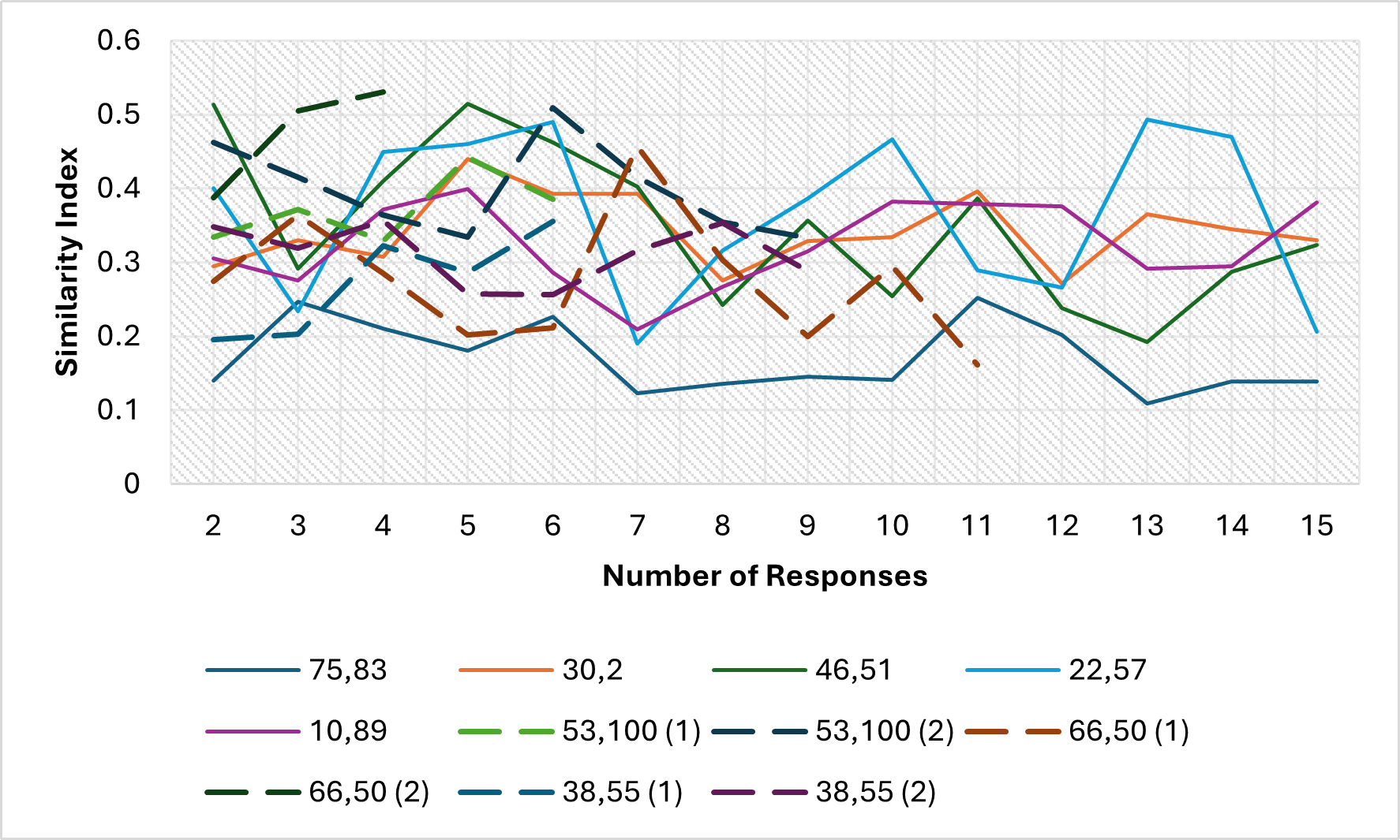

Supplement: S3 Fig — (TIF) [file pone.0322078.s005.tif]

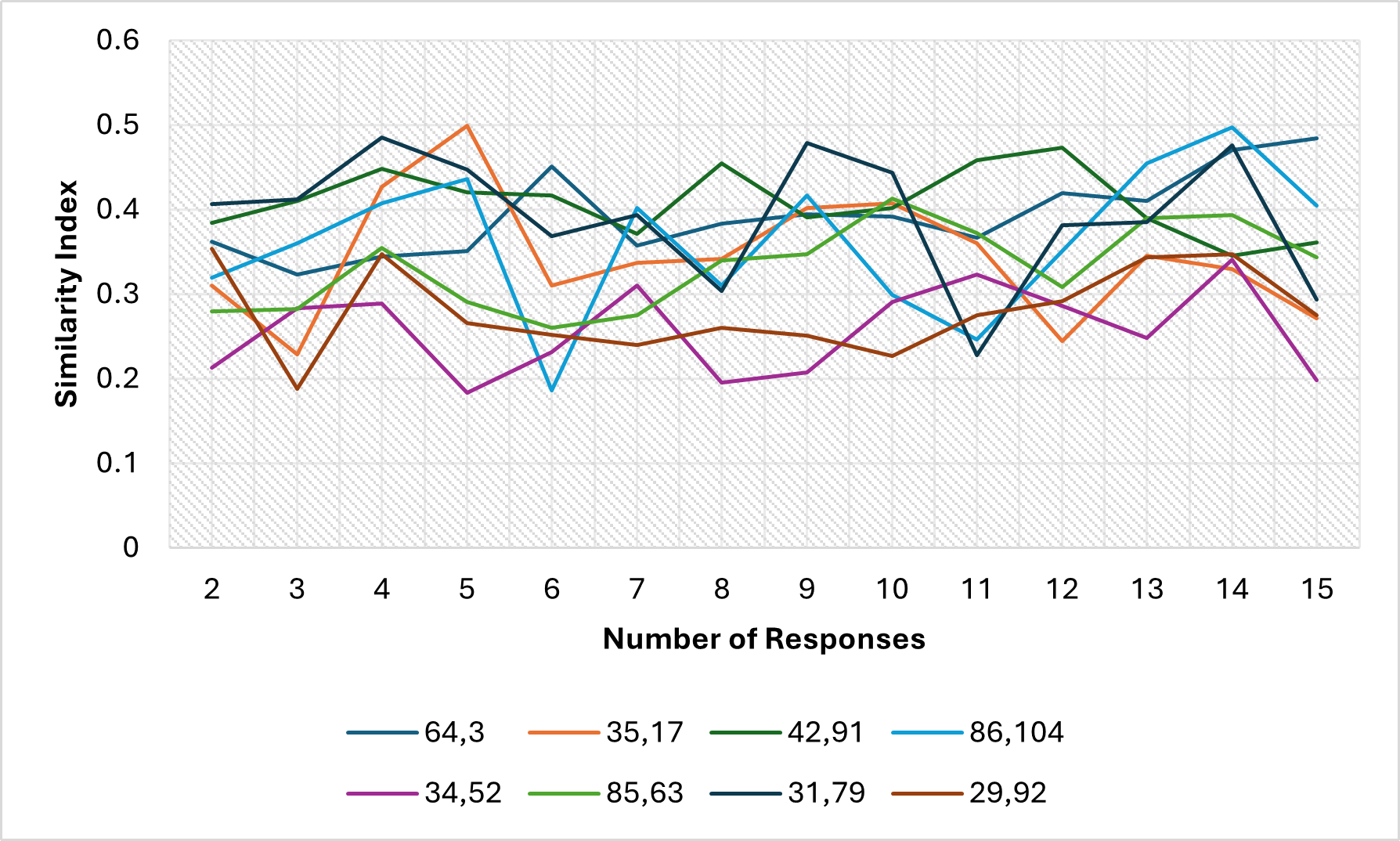

Supplement: S4 Fig — (TIF) [file pone.0322078.s006.tif]

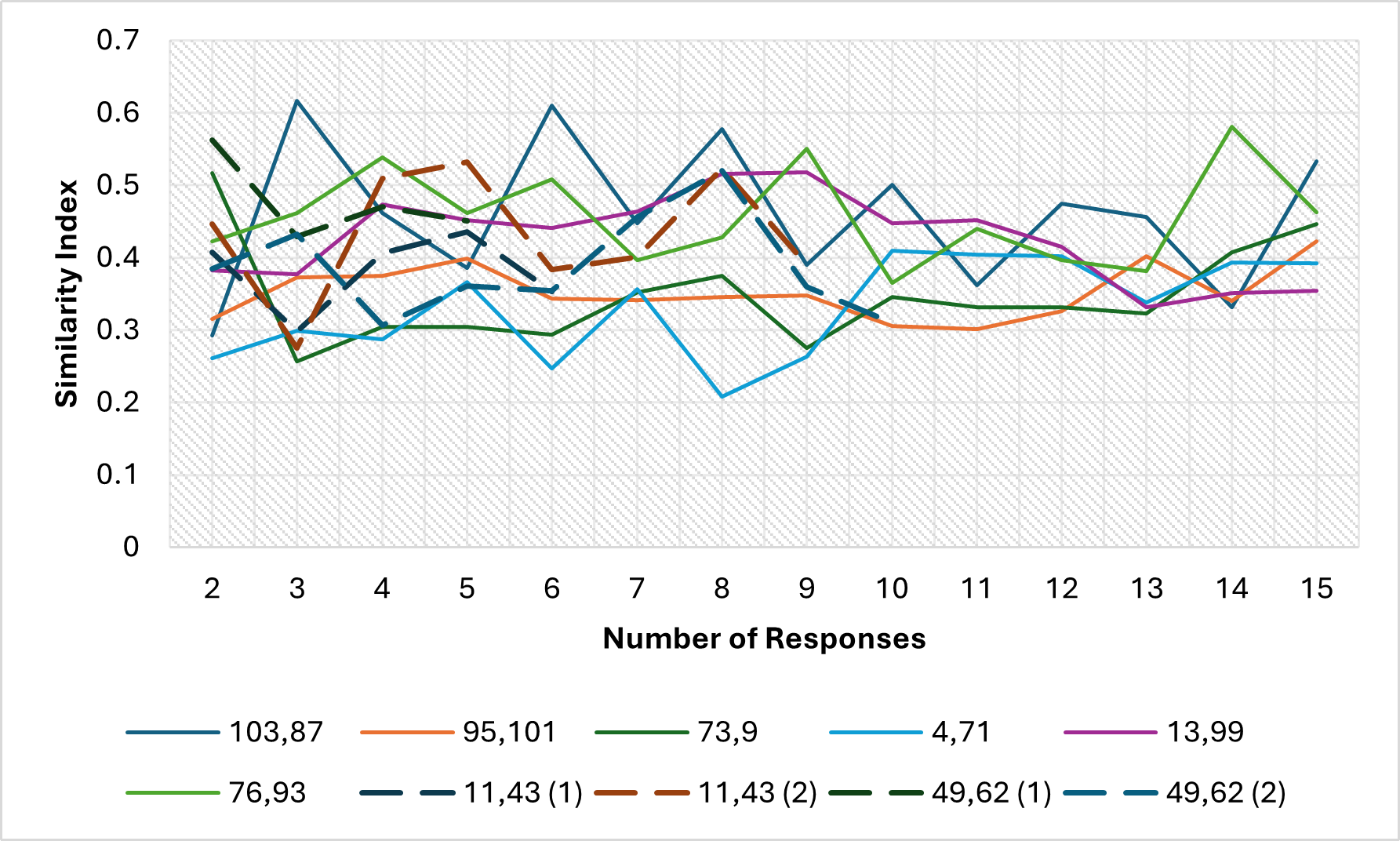

Supplement: S5 Fig — (TIF) [file pone.0322078.s007.tif]

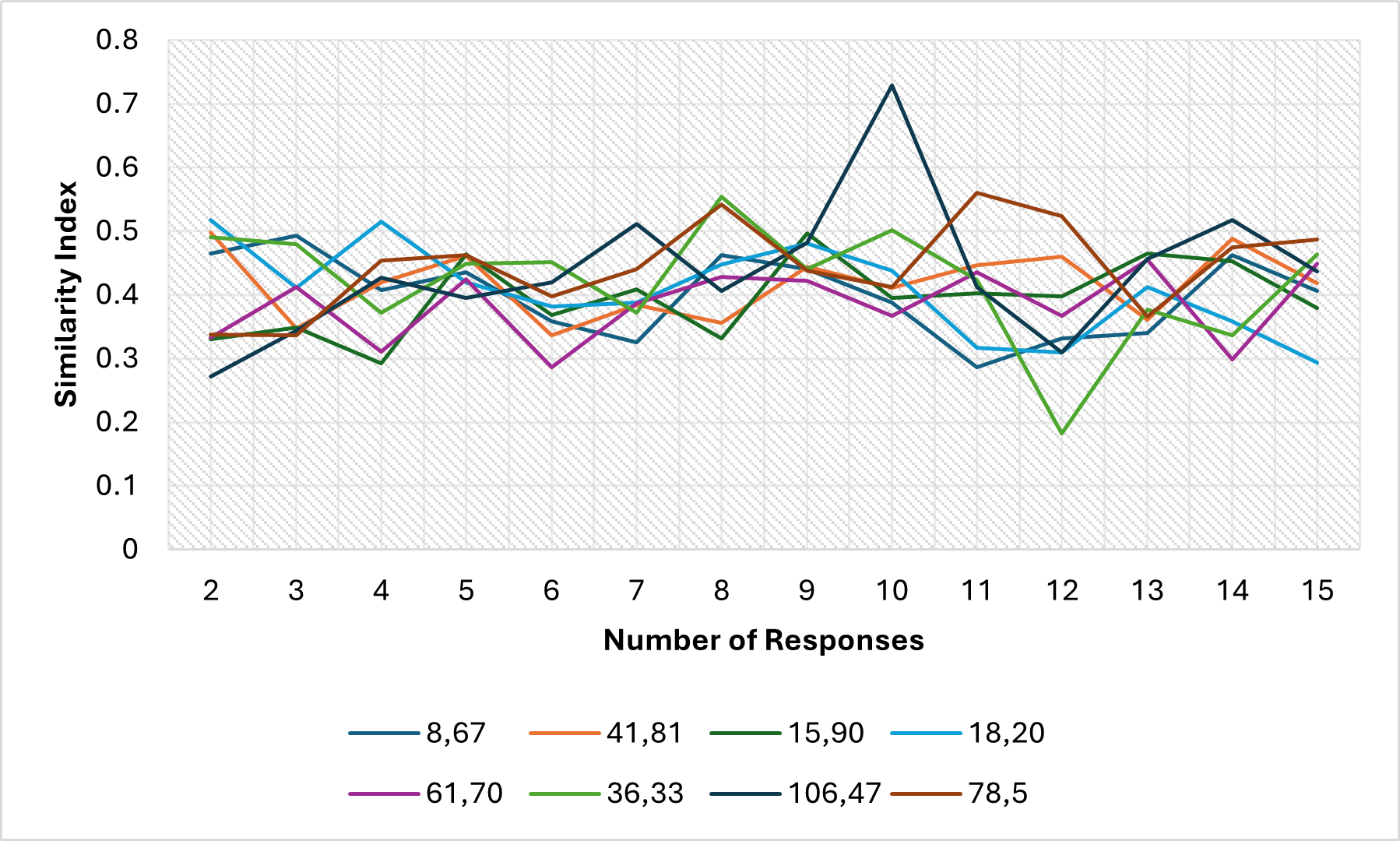

Supplement: S6 Fig — (TIF) [file pone.0322078.s008.tif]
