# Supplementary material for: Urban walkability through different lenses: A comparative study of GPT-4o and human perceptions
Source: PLoS One. 2025 Apr 29;20(4):e0322078. doi: 10.1371/journal.pone.0322078 (PMC12040139; doi:10.1371/journal.pone.0322078)
Supplement: Attachment — Submitted filename: Response to Reviewers.docx [file pone.0322078.s010.docx]

**Point-by-point Response to Reviewer’s Comments**

**Title:** Urban Walkability Through Different Lenses: A Comparative Study of GPT-4o and Human Perceptions

**Manuscript Number:** PONE-D-24-54765

**Revision Version:1**

**Revision Submission Date: 2/4/2025**

| Reviewer number |  | Reviewer comment | Author response |
| --- | --- | --- | --- |
| Associate editor | 1 | The introduction needs to be restructured by addressing the gap in literature, research problems and research aim. | Thanks for your comment.  It was rewritten. We addressed the following:   - The current tools of measuring perceived walkability (Lines 76-82). - The current use of LLMs (Lines 84-95). - Research problem and gap (Lines 97-108). |
|  | 2 | The sample size of the survey should be clarified. Providing a detailed description of the sample size is essential. | Thanks for your comment.  All were clarified in section 4.1 in Lines 332 and the lines (346-359) |
|  | 3 | The titles end with punctuation marks such as full stops and semicolons. Such marks should be removed. | Thanks for your comment.  All were removed. |
|  | 4 | The discussion section needs to add a couple of sentences that discuss the current findings from conducting a comparative study between Chat-4o and human responses.  Additionally, the research limitations of utilising qualitative study should also be discussed. | Thanks for this comment.  Regarding the first part of the comment, we had a few sentences already added in the first paragraph of the discussion. (Lines 535-537).  We added the limitations of this study and future research directions in one paragraph at the end of the discussion section (lines 609-641). Also, all the future research directions and limitations were deleted from the conclusion. |
|  | 5 | The conclusion section summarizes the central finding, but it is important to expand on suggestions for future research. | Thanks for your comment.  Suggested future research directions were added at the end of the discussion as we wanted it to be more detailed. |
|  | 6 | It is essential to review the latest article published by PLOS One to ensure the continuity of the research direction. | Thanks for your comment.  Recent related articles about walkability and LLMs were added. Check the references number [10,18,26,27,33,77]. |
|  | 7 | For compliance with PLOS One requirements, it is essential to provide the approval for human subjects research from the Institutional Review Board (IRB) or an equivalent ethics committee at Michigan State University, where the authors are affiliated. | Thanks for your comment.  The IRB number was mentioned in the third paragraph of section 3.1.1. check the lines (270-280).  Also, we have attached the IRB form in the submission. |
| Reviewer 1 | 1 | There are several unusual mistakes in the writing (e.g., line 4 of the abstract: repetitive statement of GPT-4o (GPT-4o)). Additionally, the numbering of sections and subsections is incorrect, and the conclusion section does not have a number. Moreover, the citation style is inconsistent throughout the paper; some references use a number-based format, while others follow APA. The authors must ensure consistency in the style of the paper. In general, the fluency and grammatical accuracy of the text need to be thoroughly checked. | Thanks for your comment.   - The abstract was edited. “GPT-4o” was deleted. - The numbering was fixed across the whole manuscript. - The conclusion section was numbered. |
|  | 2 | The Introduction section is too brief. It should be expanded to better contextualize the topic, clearly define the variables associated with this study, and enhance its bibliographic resources. | Thanks for your comment.  It was rewritten. |
|  | 3 | Since the first subsection of the literature review forms the core of the analysis, it would benefit from a wider range of bibliographical resources. This includes elaborating on pedestrian-friendly infrastructure, the five visual elements of walking, the perception of security, street furniture, and urban design features influencing walkable urban spaces.  Additionally, addressing the limitations of image semantic segmentation in the last paragraph of the ‘Walkability Perception’ subsection requires supporting evidence. | Thanks for your comment.  We rewrote section 2.1. For the first part of the comment, we defined walkability (lines 112-119).  Then, we illustrated the concept of perceived walkability, and we addressed a theoretical background of the main variables in studying the perceived walkability (lines 122-135). Also, we showed some current methods of measuring walkability other than image segmentation.  Regarding the second part of the comment, we presented the shortcomings of semantic segmentation methods (lines 165-164), then we showed how LLMs are more valuable than image segmentation methods (lines 165-172). |
|  | 4 | The clarity of Figure 1 must be improved. It appears to have been screenshotted while in modifiable mode, and some arrows are incomplete. Furthermore, each figure must be cited in the text and accompanied by suitable explanations. Likewise, figure captions should be described in detail. | Thanks for your comment.  Indeed, Figures 2 and 3 were screenshot as we just wanted the prompt and GPT answer. Therefore, it was hard to have them in a higher resolution. Therefore, we deleted them from the manuscript, and we think that they are no longer needed.  All of the captions were checked. |
|  | 5 | The authors need to provide information about the sample size of the conducted survey in the Methods section, along with the rationale behind the chosen number of participants. | Thanks for this comment.  The total number of sample size is 174.  In the methods, line 284, we mentioned the number of responses we had from the survey.  The rational behind chosen some responses was clarified in the lines (346-359). |
|  | 6 | Demographic data should not be presented in such a chaotic manner. It would be more effective to summarize the participation range of each group using percentages to enhance the fluency of the text. | Thanks for your comment.  We rewrote the whole paragraph. Check the lines (332-344). |
|  | 7 | One of the most significant limitations of this study is that some of the indicators of walkability mentioned (e.g., livability and accessibility) may not be measurable using the current tools. This limitation should be explicitly noted in the Conclusion section. Additionally, the suggestions for further research should be improved by elaborating on other potential applications of AI in interpreting, organizing, and analyzing urban planning research. | Thanks for your comment.  We decided to write one detailed paragraph at the of the discussion to show all the study limitations and future research.  This was added to the last paragraph of the discussion. Check the lines (609-641). |
| Reviewer 2 | 1 | The overall quality of the images in the manuscript is suboptimal. There are several areas where the visuals could be improved for better clarity and more precise communication. For example, in Figure 1, the arrows below the image are unclear regarding what they are pointing to or connecting. Additionally, the image appears to have been captured as a screenshot, which is not ideal for a scientific publication. This screenshot retains an active selection box. | Thanks for this comment.  Indeed, Figures 2 and 3 were screenshot as we just wanted the prompt and GPT answer. Therefore, it was hard to have them in a higher resolution. Therefore, we deleted them from the manuscript, and we think that they are no longer needed. |
|  | 2 | Section 2 Methodology: The description of how images were selected for evaluation is unclear. It is crucial to elaborate on the selection criteria for images and the participant demographics (e.g., age, profession, cultural background) to ensure the generalizability of results.  It would be helpful to mention the number of human participants and the diversity of the urban areas assessed. More specific details on the data (e.g., demographic diversity of participants or add more showcase of cityscape images to better illustrate) would strengthen the findings. | Thanks for your comment.   - To elaborate the selection criteria of the images, the second paragraph of section 3.1.1 was edited. check the lines (239-253 - The participants were **randomly selected** through an **online survey platform**, which enabled us to reach a diverse audience without targeting specific demographics. Check the lines (282-291). Also, the numbers and the demographics of participants were mentioned in section 4.1. check the lines (332-344). |
|  | 3 | In the result section, the manuscript presents similarity scores (e.g., 0.4575) without sufficient explanation of how these scores were calculated, their statistical significance, or what constitutes a significant alignment between GPT-4o and human evaluations. The concept of a "coherence score" is not mentioned or defined, which may be critical for understanding the reliability and meaningfulness of the results.  Suggestion: 1. Provide a detailed explanation of how the similarity scores were computed, including the citations, equations, or models used to derive these values.  2. Clarify the significance of the similarity scores in the context of human and AI evaluation alignment. For example, what threshold value of the score represents a significant alignment between GPT-4o and human responses? Are there statistical tests that were performed to validate these scores?  3. If "coherence score" is a part of the analysis, please define it explicitly and explain its role in assessing the results. | Thanks for your comment.  We edited the first paragraph of section 4.5.1. The suggestions were added. We explained the metrics, threshold and all the references needed. Please, check the lines (429-439).  Thanks for your suggestions. |
|  | 4 | The paper mentions the potential of AI to assist in urban planning tasks but stops short of providing a clear set of recommendations for the practical implementation of AI in urban design. The study could benefit from a deeper discussion of how AI-based assessments of urban environments could influence urban planning. Are there specific applications where GPT-4o might be more useful? What are the limitations for human-cantered decisions?  For example, how do cultural or personal factors influence human judgments of walkability? How might GPT-4o handle these subjective differences? | Thanks for the comments and suggestions.  Those aspects were added up and discussed at the end of the discussion section. Please, check the lines (609-641). |
|  | 5 | There is a minor error of the section serial number in the manuscript, where Section 2 is followed directly by Section 4, without Section 3 being included. | Thanks for your comment.  The numbering was fixed. |
